# Can We Rely on Mobile Devices and Other Gadgets to Assess the Postural Balance of Healthy Individuals? A Systematic Review

**DOI:** 10.3390/s19132972

**Published:** 2019-07-05

**Authors:** Alexandre S. Pinho, Ana P. Salazar, Ewald M. Hennig, Barbara C. Spessato, Antoinette Domingo, Aline S. Pagnussat

**Affiliations:** 1 Movement Analysis and Rehabilitation Laboratory, Universidade Federal de Ciências da Saúde de Porto Alegre (UFCSPA), Porto Alegre, RS 90050-170, Brazil; 2 Health Sciences Graduate Program, Universidade Federal de Ciências da Saúde de Porto Alegre (UFCSPA), Porto Alegre, RS 90050-170, Brazil; 3 Rehabilitation Sciences Graduate Program, Universidade Federal de Ciências da Saúde de Porto Alegre (UFCSPA), Porto Alegre, RS 90050-170, Brazil; 4 Institute of Health & Biomedical Innovation (IHBI), Queensland University of Technology (QUT), Kelvin Grove, Brisbane QLD 4059, Australia; 5 School of Exercise and Nutritional Sciences, San Diego State University, San Diego, CA 92182-7251, USA

**Keywords:** mHealth, postural balance, wearable electronic devices, mobile applications

## Abstract

The consequences of falls, costs, and complexity of conventional evaluation protocols have motivated researchers to develop more effective balance assessments tools. Healthcare practitioners are incorporating the use of mobile phones and other gadgets (smartphones and tablets) to enhance accessibility in balance evaluations with reasonable sensitivity and good cost–benefit. The prospects are evident, as well as the need to identify weakness and highlight the strengths of the different approaches. In order to verify if mobile devices and other gadgets are able to assess balance, four electronic databases were searched from their inception to February 2019. Studies reporting the use of inertial sensors on mobile and other gadgets to assess balance in healthy adults, compared to other evaluation methods were included. The quality of the nine studies selected was assessed and the current protocols often used were summarized. Most studies did not provide enough information about their assessment protocols, limiting the reproducibility and the reliability of the results. Data gathered from the studies did not allow us to conclude if mobile devices and other gadgets have discriminatory power (accuracy) to assess postural balance. Although the approach is promising, the overall quality of the available studies is low to moderate.

## 1. Introduction

According to the Global Health Organization, falls are the second leading cause of deaths due to accidents or unintentional injury worldwide [1]. The consequences of falls, especially in the elderly population, have drawn attention to the development of fall prevention strategies, focusing on training protocols and more effective/precise balance assessments [1,2,3,4].

A wide variety of mathematical models, evaluation protocols, and instruments have been proposed for quantitative measurements of balance. The costs, and complexity of devices for quantitative data make, the assessment and interpretation of results challenging and restricted to academic research or expensive private services [5,6]. Recently, healthcare practitioners have been incorporating the use of less expensive sensors for balance assessment. Mobile phones and other gadgets (smartphones and tablets) have been used because they have triaxial accelerometers and gyroscopes embedded, which turn them into wireless inertial measurement units (IMU). Although these devices have dramatically improved regarding their speed of real-time computing processing and accuracy [7,8,9], there are still some challenges to overcome. For instance, it is not well understood how this data can be best interpreted and applied to clinical practice [10,11].

Mobile sensors and processing apps (applications/software) are novel technologies used to enhance accessibility to balance evaluations with reasonable sensitivity and good cost–benefit [12,13,14,15,16,17,18]. This technology allows us to assess balance through acceleration measurement resultants calculated using a simplified approach from the position of the center of mass (COM) usually described by an arbitrary (not estimated) single point where the sensor is positioned [19,20]. Some advantages of using inertial sensors from smartphones or tablets to assess balance are: (1) The equipment is affordable and accessible, (2) allows real-time evaluations, (3) self-administered protocols, (4) quick and reliable feedback, (5) user-friendly apps and charts reports, (6) easy disseminating results, improving the link between patient, healthcare professionals, and family, (7) ease of understanding and monitoring follow-up [9].

As a disadvantage, we can point out the nature of Micro-Electro-Mechanical Systems (MEMS) sensing technology embedded on those devices, which addresses some intrinsic errors to data acquisition, mostly regarding deterministic errors. The main source of these errors is “white noise”. These problems may be overcome, in theory, by carefully analyzing data and using specific filters and proper calibration [21]. Smartphone ownership is on the rise in emerging economies, but its cost is still an issue. The global median rate is 59%, but it could be as high as 94% in South Korea, 83% in Israel, 82% in Australia. On the other hand, this rate is reported to be less than 50% in 12 of 22 countries surveyed by the Pew Research Center (2018) or even less in poorer countries [22].

Even though the use of mobile phones and other gadgets with built-in sensors are not fully validated, the prospects are evident as well as the need to question weakness and strengths of the different approaches [4,8,9]. The primary outcome of this study was to verify if mobile devices and other gadgets are able to assess balance. The secondary outcomes were, to review the current protocols used to assess balance with consumer-level mobile devices (mobile phones and tablets) and to summarize: (a) parameters used to define balance, (b) main characteristics and technical specifications of devices and sensors, (c) mathematical models and algorithms used to process data. Additionally, we examined the potentialities and limitations of protocols to guide readers about the most reliable and convenient method of accelerometer-based balance assessment.

## 2. Materials and Methods

This systematic review was reported according to the preferred reporting items for systematic reviews and meta-analysis (PRISMA) and Cochrane guidelines [23,24]. The protocol was recorded at the International Prospective Register of Systematic Reviews (PROSPERO, CRD42018103481).

### 2.1. Eligibility and Inclusion Criteria

We included only articles that reported the comparison of general balance evaluation methods to the use of mobile inertial sensors as devices (smartphones and tablets). Regardless of the methods for blinding and randomization, all study designs were included if they assessed standing balance in healthy adults and had been published up to 2019.

### 2.2. Search Strategy

A systematic search was conducted (from inception to February 2019) using the following databases: PubMed, EMBASE, Scopus, and Cochrane Central. The search strategy included terms as ‘accelerometry,’ ‘accelerometer,’ ‘gyroscope,’ ‘body wear sensors,’ ‘wearable sensors,’ ‘inertial sensors’, ‘IMU’, ‘inertial measurement units’, ‘mobile application’, ‘mobile app’, ‘mobile device’, ‘smartphone app’ and words related to ‘postural balance,’ ‘sway,’ or ‘postural control.’ The search was limited to papers written in English, Spanish, and Portuguese with no restriction to date. The complete search strategy is presented in Appendix A. (available as supplementary material online)

### 2.3. Data Extraction, Risk of Bias and Quality Assessment

Two reviewers (ASP and APS) independently screened the studies by titles and abstracts and deleted duplicates based on the inclusion criteria. After this step, the same reviewers assessed the full texts separately. The authors were contacted by email when data were not available. If the two reviewers did not find a consensus in all phases of the selection (including the screening for the quality assessment), a third reviewer (BCS) made the final decision. All the reviewers have broad experience in the research field. EndNoteTM X7 software (Clarivate Analytics US LLC, Philadelphia, PA, USA) was used to select and search for articles. During the screening step, the selection was blinded, and there was no disagreement between ASP and APS. The data extracted from the included studies were: Type of study, number of participants, type and location of the wearable sensor, time acquisition, general conditions of assessments, and primary outcomes of each study. If necessary, a third reviewer was consulted to solve disagreements.

Methodological quality assessments were performed for all studies using the Quality Assessment Tool for Observational Cohort and Cross-Sectional Studies from the National Heart, Lung and Blood Institute (NHLBI) of the United States National Institutes of Health (NIH) [25]. A fourteen-criteria tool designed for a critical appraisal which involves considering the risk of potential for selection bias. This instrument measures the ability of the study to draw associative conclusions about the effects of the exposures being studied on outcomes. The quality was expressed as a percentage of the total possible score, with a maximum of two points for each criterion (“Yes” = 2, “Cannot determine” = 1, “No” = 0). The studies were classified as: “high quality” (>75%), “moderate quality” (>50% to 75%), “low quality” (25% to 50%), and “very low quality” (<25%). Considering the characteristics of papers included in this review, only 12 items were evaluated with a maximum of 24 points (available as supplementary material online, Appendix B).

Due to the lack of a particular tool to access the consistency of the balance protocols information, we created a 10-point checklist to access the main information on balance protocols addressing aspects related to measurement bias and reproducibility. Even if this tool did not have its efficacy validated yet, we believe that due to its custom developed characteristics, it brings light whether the main parameters that can possibly influence the results of the balance assessment in general were present or not (available as supplementary material online, Appendix C). For each topic, two researchers gave a yes (Y), or no (N) score and the sum of all topics resulted in the paper’s total score (when a topic was “not applicable” a “Y” was given). If the study scored 8 to 10 points, we classified it as being “highly detailed.” In other words, a “highly detailed” study presents great consistency, low risk of measurement bias, and enough information to allow reproducibility. If the study was scored between six and seven, we classified it as “fairly detailed,” or, the study has some risk of measurement bias but is consistent enough to allow reproducibility. Finally, a study with less than six points, the study was classified as “poorly detailed”, or with high risk of measurement bias and/or not fully reproducible (available as supplementary material online, Appendix C).

## 3. Results

**Figure 1:**
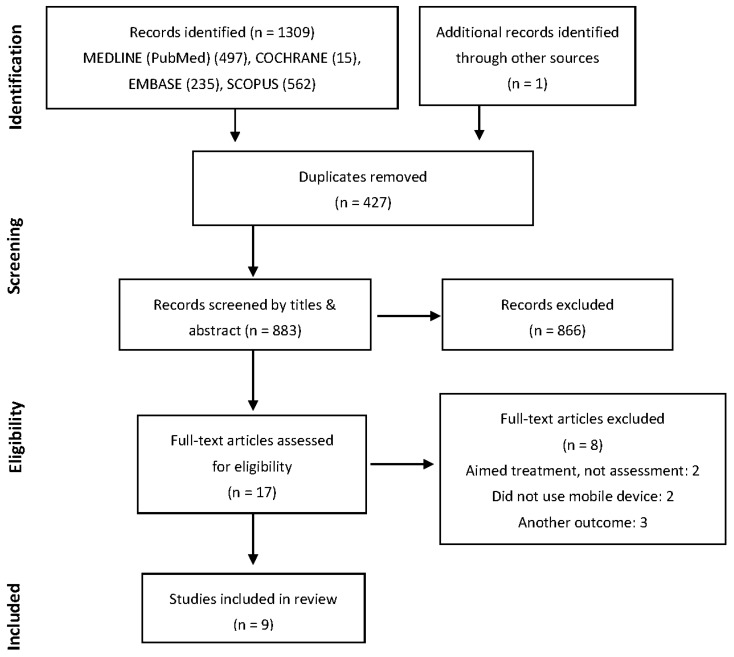
Flow diagram.

The initial search identified 1309 studies. After excluding duplicates (427) and screening titles and abstracts, nine papers were considered potentially relevant and were included in this systematic review. All studies included healthy individuals [14,15,17,18,26,27,28,29,30] and had a cross-sectional design and have been published between 2014 and 2019. A flow diagram elucidating the study selection is provided in Figure 1.

### 3.1. Sample Characteristics

The sample size varied from 12 to 60 individuals. Studies included males and females, from different age groups between 16.4 and 78.9 years. Five studies included only young adults and teenagers. One study categorized subjects into three age groups, young, middle age, and older adults [14] and other study selected only the older population [15]. One study did not report the age of the subjects [29], and others did not present the standard deviation of their sample [27]. Four papers did not report height and body mass of the included subjects [14,15,27,29]. Hsieh et al. selected their sample classifying between high risk and low risk of falls [30]. Table 1 shows the sample characteristics of the studies.

### 3.2. Overview of Studies Objectives

All studies had a cross-sectional design and used dedicated apps [15,17,18,28,29] or raw data acquisition apps to determine the capability to evaluate postural balance [14,26,27]. One study did not report this information [30]. Two studies compared the data acquired using gadgets to “gold standard” balance assessment devices: Biodex Balance System™ [28] or NeuroCom^®^ Smart Balance Master [26]. Four other studies compared gadgets with kinematic data by motion capture system [15,27], commercial accelerometers [14], and force plates [30]. Subjective clinical evaluation tests (full or adapted versions) were performed in five papers [15,17,27,28,29]. One paper performed the Physiological Profile Assessment (PPA) test on their participants [30]. The PPA test measures fall risk based on vision, reaction time, leg strength, proprioception, and balance, which gives a score and characterizes individuals between low risk and high risk of fall [31]. The iOS (Apple Inc. Cupertino, CA, USA) was the operating system of seven studies [14,15,17,26,27,28,29], and the Android Inc. (by Google Inc. Palo Alto, CA, USA) were at two studies [18,30].

### 3.3. Balance Assessment Protocols

Tasks used to evaluate postural balance varied across the studies and included: Balance error scoring system (BESS) [15,17,27], athlete single leg test [28], Romberg and tandem Romberg tests [29], NeuroCom^®^ sensory organization test (SOT) [27], SWAY balance test [17,28], and other general tasks [14,18,30], (Table 2).

One paper used the six formal conditions of BESS [27], which is performed with eyes closed for 20 s: (1) Double-leg stance, firm surface, (2) single-leg stance, firm surface, (3) tandem stance, firm surface (dominant leg in front of the other), (4) double-leg stance, foam surface, (5) double-leg stance, foam surface, and (6) tandem stance, foam surface. Patterson et al. 2014 [17] adapted the BESS by modifying the hand’s position during the test. Lastly, one study altered the BESS conditions adjusting the test to an older population [15]. The author modified the analysis by excluding the single-leg stance, performing some parts of the test with open eyes.

One study followed the NeuroCom^®^ protocol device [26], which uses the NeuroCom^®^ sensory organization test (SOT), resulting in an equilibrium score. The protocol includes several procedures that combine stable and unstable surface with open and closed eyes, as well as with an oscillation of the visual references. The authors evaluated 49 individuals through Neurocom to determine whether an accelerometer and gyroscope data sampled from a consumer electronics device (iPad2) could provide enough resolution of the center of gravity (COG) movements to accurately quantify postural stability. Six conditions of SOT were used to compare the scores generated and calculated from both devices. Limits of agreement were defined as the mean bias (NeuroCom, iPad) + 2 standard deviations. Through the comparison of the real-time center of gravity sway, they found that the best agreement by the mean difference in equilibrium scores was of 0.01% for the SOT-1 and the largest difference was −6.2% for the SOT-5.

Two other papers performed the SWAY Balance Test [17,28]. This test consists of five stances including single leg stance, feet together and tandem during 10 s on a firm surface with eyes closed. One article evaluated 30 young individuals performing a single trial of the Athlete Single Leg Test requesting the subjects to stand on their non-dominant foot for 10 s [28]. Balance scores were generated from arbitrary units of both systems determined by undisclosed calculations. The balance scores derived from the smartphone accelerometers (SWAY Balance Mobile Application software) were consistent with balance scores obtained from the Biodex System, showing no significant differences (*p* = 0.818) between the means. A significant correlation between the two data sets was found (*p* < 0.01, r = 0.632).

Other tasks chosen by authors included a dual-task protocol with a “letter fluency test” in a parallel stance and a semi-tandem stance with eyes open and closed [14] and with a concurrent cognitive challenge, having the participants simultaneously subtracting by seven from a random number between 100 and 200 [30]. Eight different conditions were used with the myAnkle application and are detailed in Table 1 and Table 2 [18]. One paper used the *Romberg test and the Romberg tandem test* performed with and without noise restriction. Subjects went through a combination of sixteen postures, including open and closed eyes, feet together, and tandem, on a firm and foam surface [29].

#### 3.3.1. Feet and Arms Position

**Figure 2:**
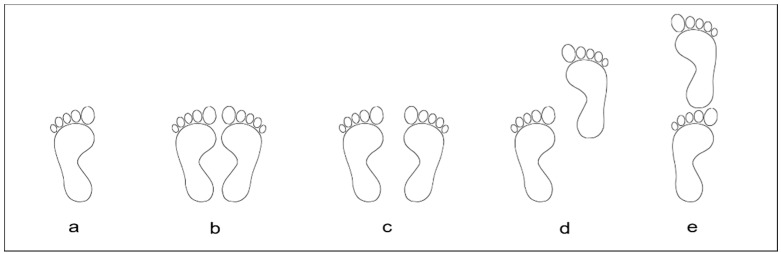
Feet positions: a = Single leg, b = Feet together, c = Feet apart, d = Semi-tandem, e = Tandem3.3.2. Visual Reference

Regarding foot position, some papers followed closed protocols [15,17,26,27]. Other studies evaluated only non-dominant single leg stance [28], feet parallel and semi-tandem [14], tandem, feet closed together and apart [18], or feet together and tandem [29] (Figure 2). Studies used a barefoot condition [15,18,27] or assessed subjects wearing socks [26] or shoes not specifying the type [17]. Four studies did not describe foot condition [14,28,29], and one study only described it partially [30].

Regarding the arms or hands position, three articles used a software application protocol where subjects held the mobile at the sternum mid-point [17,28,30]. Three papers described the position of the “hands” instead of “arms”, which were resting on the subjects’ iliac crests [15,27] or on the subject’s hips [18]. Authors also instructed subjects to “rest the arms at body side” according to the device’s protocol of SOT [26] and to use the same “arms position of Romberg’s tests” [29]. One paper did not specify this information [14].

Two studies described a visual target reference during the mobile data acquisition which was located at 3 m [15] and 4 m [18] ahead but did not mention the height from the ground and size of the target. Four authors did not report visual reference. In other studies, this aspect could not be analyzed due to a closed eyes condition [17,27] or some specific visual task [26].

#### 3.3.2. Number of Acquisitions, Sessions and Total Time of Acquisition

Most studies conducted only one trial of data acquisition [14,17,18,27,28,29] while others performed three [26] and two trials [15,30]. The time acquisition ranged between 10 s and 60 s. None of the articles used or reported using a time window (cropped time) at the analysis (Table 3). Three studies used a well-established sampling rate recommendation of 100 Hz [10] for balance data acquisition [15,26,27]. In three other studies, the rates varied from 200 Hz [30], 88–92 Hz [14] to 14–15 Hz [18]. Three articles did not fully describe the sampling rates [17,28,29] (Table 3).

Only one study presented test–retest reliability. The author repeated data acquisition twice. Although the description indicates that the test–retest was within the same day with a short interval, there was no time interval reported between acquisitions [14]. The intraclass correlation coefficient (ICC) values found for the root mean square (RMS) of the accelerations was 0.83 and 0.90, and for the Sway Area, ICC was 0.81 and 0.91 during parallel stance and semi-tandem stance respectively.

#### 3.3.3. Measurement Device and Position

**Figure 3:**
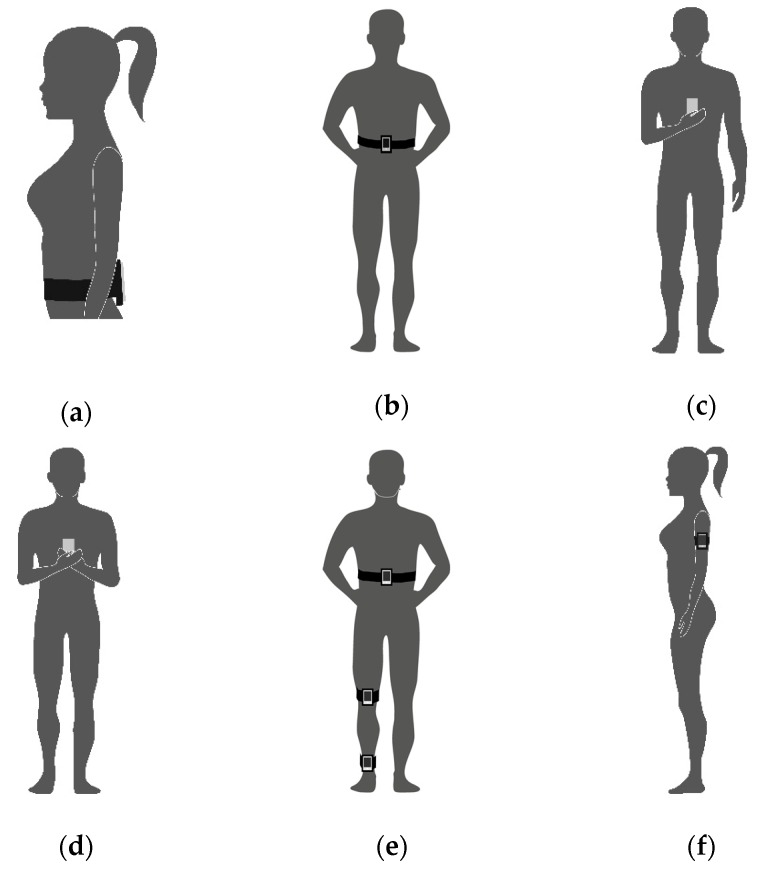
Devices and arms positions: (**a**) Lumbar or sacral region arms not reported [14,26]; (**b**) Lumbar or sacral region [15,27]; (**c**) Sternum dominated hand [30]; (**d**) Sternum both hands [17,28] (**e**) Malleolus, patella, umbilicus [18] (**f**) left upper arm [29].

Studies used different wearable sensors (Table 2). Three studies used iPad devices [15,26,27], three used iPods [14,17,28] and three used smartphones, an iPhone [29], a LG Optimus One [18] and a Samsung Galaxy S6 [30]. Four studies reported placing the mobile sensor on the participants’ lumbar or sacral region [14,15,26,27]. Three studies placed the gadget on the sternal midpoint [17,28,30], one on the left upper arm [29] and another one positioned three devices on different body places (malleolus, patella, umbilicus) [18] (Figure 3).

#### 3.3.4. Devices Synchronization

Acquisitions of data with the use of more than one piece of equipment in which the time phases must occur at the same time, theoretically presuppose the use of a synchronization method. Three studies did not report any synchronization method [18,28,30], while four studies adequately described this process [14,15,26,27]. In two articles, the synchronization procedure did not apply [17,29].

#### 3.3.5. Measurements and Signal Processing Parameters

The primary signal processing parameters used in quantitative continuous data measurements are briefly listed (available as supplementary material online, Appendix D) as stated in the study. Some studies reported using the raw data to run their own post-processing algorithms for the computing balance metrics [14,15,26,27,30]. One author designed a mobile phone app [18]. Other authors did not report if they had access to the app algorithm or calculations [17,28,29].

### 3.4. Methodological Quality Assessment

#### 3.4.1. Quality Assessment Tool for Observational Cohort and Cross-Sectional Studies NIH-NHLBI

Three papers were classified as “low quality” (from 25% to 50%) [17,28,29] and six papers as “moderate quality” (from >50% to 75%) [14,15,18,26,27,30]. No articles were considered “very low quality” (<25%) nor “high quality” (>75%), (available as supplementary material online, Appendix B).

#### 3.4.2. A 10-Point Checklist for Balance Assessment Protocols

Four studies were classified as “highly detailed” [15,18,26,27]. One study was considered “fairly detailed” [17], and four studies were considered “poorly detailed” [14,28,29,30] (available as supplementary material online, Appendix C).

## 4. Discussion

This study aimed to review systematically the current protocols used to assess balance with mobile devices. We provided an overview of parameters used to define balance, main characteristics of devices and technical specifications, mathematical models, and algorithms used to process data. Briefly, we found that studies presented good consistency with the assessment procedures. However, we found a widespread lack of standardization in data acquisition, which compromises the data repeatability and reproducibility. Besides, methods to evaluate the mobile capability in assessing balance were too varied among studies, as well as the mathematical models, variables, tasks, and posture conditions.

It is well known that methodological aspects like anthropometric characteristics, time of acquisition, feet and arms position can influence the results and the reliability of measurements of postural balance. So, these parameters must be controlled and described in detail in scientific papers as it has been already established in the literature [10,20,32,33]. Five articles did not report height and body mass data [14,15,27,29,30]. Normalization methods for a proper comparison among subjects were reported by a few studies [15,27]. Height and body mass are an essential anthropometric characteristic affecting the base of support and the COM position, thus, these parameters must be controlled or normalized while comparing groups or describing samples to assess balance. For individual assessments by clinicians or customers, this issue could be of less importance, considering these parameters are less susceptible to changes.

Another aspect to be considered in balance evaluation protocols is the time of acquisition. Time of acquisition ranged from 10 s to 60 s in the included papers. The literature describes that time of acquisition may result in slight changes in balance parameters [32]. However, the shorter the time acquisition, the higher the synchronization control of devices must be, which was not a point of concern for all authors. We highlight the time of acquisition as another aspect that influence decisions about mathematical models and data processing methods [32].

Feet and arms positions are directly related to the physics concepts of stability. Moving the feet apart increases the size of the base of support and the capacity of stabilizing, as evidenced by patterns of the center of pressure (COP) variables [34]. On the other hand, the position of arms can alter the body oscillations and stability by slight changes in the COM affecting the base of support. This is another critical point to be considered. The studies included in this review used a wide range of feet and arms positions, and most studies used different restricted postures during the balance assessment. The body oscillation is changed when an individual is restricted or not to a specific posture. So, the data acquired from different protocols may be diverse, not allowing comparison, or not reflecting the general characteristics of balance in some cases.

Based on the physics concepts, the best way to describe balance and its displacement remains an open question [35,36,37,38,39]. It is very common to use the COM sway represented, estimated as a single point around the base of the lumbar spine [37]. Another possibility is to use the COP trajectories, which represent a weighted average of all the pressures over the surface at the base of support [35,36,38,39]. These two parameters are measured by different techniques and the position of the sensors can influence the results. The majority of studies positioned the sensors on the pelvis, lumbar, and sacral vertebra [14,15,26,27]. Some studies have chosen the upper limbs [29], lower limbs [18], and chest [17,28,30] to place the sensors. The trunk seems to be the best option due to the proximity of the body COM as well as avoiding unwanted movements of limbs interfering with balance assessment. A previous study showed good to excellent test–retest reliability using acceleration rates and COP parameters when the sensor is placed in the lumbar region [12], reinforcing this statement.

We cannot determine if the best choice is to fix the device in some specific area of the body or just ask for the individual to hold the device on their own. Encouraging the individual to hold the device would favor the self-administration of balance tests and empower patients to care about their health but could compromise data acquisition. Positioning near or away from the body’s center of mass will influence the movement degree of freedom caused by specific joints strategies for balance control [12]. The choice of the device position would affect the relative plane orientation and influence the repeatability and data accuracy. Although there is a lack of studies covering those aspects, it is known that the design of applications and decisions on protocol procedures induce specific and careful data processing. Moreover, the algorithm must be in conformity with the theoretical approach [8].

One of the major concerns in protocols of balance evaluation is the time acquisition. In studies included in this review, time acquisition ranged from 10 to 60 seconds. The shorter the time interval chosen, the more caution measures had to be taken due to the accuracy needed for synchronizing the devices. Additionally, the time of acquisition critically influence the decisions on the mathematical model and data processing methods [32]. Selecting a time window (cropped time window) is a usual procedure in quantitative balance analysis when a few seconds are withdrawn from the total acquisition time (arbitrarily) at the beginning and at the end of each attempt. Although not clear in the literature, it has been justified by reducing disturbing movements in the initial posture and attenuating the effects of fatigue, ensuring steadiness with less unwanted "noise" and "artifacts" in the signal. None of the papers reported using this method. The main objective of the studies selected was a comparison between sensors and devices, what probably dispense the use of this procedure, but raises a doubt whether it would increase or not the sensitivity of the protocols and data correlation.

A test–retest approach might have also enhanced the results, which was performed only by one study [14]. Likewise, the number of acquisitions, although a previous study which compared the acceleration data to the center of pressure reported that the data from three trials are similar to those obtained in only one trial [12]. Even being a signal with stochastics characteristics, it is suggested that only one trial may be reliable and useful to be applied in clinical practice.

Signal processing methods varied among studies and included the calculation of COM, COP, and raw acceleration through the measurements of RMS, the standard deviation, the maximum peak of displacement, maximum amplitude displacement, and sway area. All of these parameters can be applied in balance assessments [40]. One study used the raw acceleration data as a parameter to define stability, which is not a direct measurement of the position and is an unusual method to describe stability. A previous systematic review explored the best outcomes to assess standing balance and walking stability in subjects with Parkinson’s disease. The authors included 26 studies and defined “jerk” (the time derivative of acceleration) and trunk RMS acceleration as the most useful measures to differentiate patients from healthy controls [41].

It is important to highlight the use of two “gold standard” clinical devices to evaluate young individuals. One aimed a validation of measurement with a specific mobile software [28] concluding that the scores from the smartphone were consistent with the validated balance system. The other compared equilibrium scores [26] calculating the limits of agreement between the devices. The author concludes that mobile hardware provided data of sufficient precision and accuracy to quantify postural stability is a reasonable approach for in clinical and field environments. At the Quality Assessment Tool for Observational Cohort and Cross-Sectional Studies NIH-NHLBI, the studies ware ranked as “low quality” [28] and “moderate quality” [26], respectively. At the 10-point checklist for balance assessment protocols, they achieved “poorly detailed” [28] and “highly detailed” [26], respectively.

This systematic review presents some limitations that make challenging to state recommendations about the most appropriate protocol to assess balance using gadgets. The majority of studies included in this review did not provide sufficient information about their assessment protocols, which make difficult the reproducibility of the evaluation, the reliability of the results and limiting the judgment of the discriminatory power (accuracy) of studies to assess postural balance. The overall quality of studies included in this review was low to moderate using the methodological Quality Assessment Tool for Observational Cohort and Cross-Sectional Studies from the NHLBI, NIH weakening the consistency of the conclusions from the studies due to the lack of information on the internal and external validity and possible increase of risk of bias. It is also important to state that the 10-point checklist for balance assessment protocols used to assess the studies in this review is a custom developed tool and is not validated for its efficacy, although it was created based upon authors expertise and after a detailed discussion of the methods presented.

Considering the quality of the evaluation procedures, technical specifications, and data processing information, only four studies were classified as “highly detailed” [15,18,26,27], restricting the reproducibility of the protocols. Finally, most studies lack a direct sensor comparison, using a "gold standard" transducer system to determine the accuracy of the various transducer outputs from mobile devices, a question that still has to be addressed.

## 5. Conclusions

The results from this systematic review did not allow to perform an evaluation of the diagnostic and accuracy tests as expected. Thus, from our preliminary findings, we cannot ensure the use of mobile devices and other gadgets to assess postural balance. However, two studies presented consistent data supporting enough accuracy and good reliability for the use of this method to evaluate healthy young individuals. Due to the differences in hardware and operating systems, the comparisons between several mobile phone systems that are currently on the market is still a fragile aspect that needs to be explored. Clear balance protocol information, anthropometric characteristics of the sample, and technical specifications of the equipment and sensors are indispensable and have to be stated. Further studies are highly encouraged, with adequate sample size, different population, test–retest measurements, and low risk of bias are necessary to provide a better understanding of this promising approach.

## Figures and Tables

**Table 1:** Sample demographic characteristics (mean ± standard deviation).

Author	Sample Gender	Age Years	Height (cm) Body Mass (kg)
Alberts et al., 2015 [26]	n = 49 22 male	19.5 ± 3.1	167.7 ± 13.2 68.5 ± 17.5
Alberts et al., 2015 [27]	n = 32 14 male	20.9 ± NR	NR
Kosse et al., 2015 [14]	n = 60 28 male	26 ± 3.9 (young) 45 ± 6.7 (middle) 65 ± 5.5 (older)	NR
Hsieh et al., 2019 [30]	n = 30 12 male	64.8 ± 4.5 (Low RF) 72.3 ± 6.6 (High RF)	NR
Ozinga et al., 2014 [15]	n = 12 5 male	68.3 ± 6.9	NR
Patterson et al., 2014 [17]	n = 21 7 male	23 ± 3.34	171.66 ± 10.2 82.76 ± 25.69
Patterson et al., 2014 [28]	n = 30 13 male	26.1 ± 8.5	170,1 ± 7,9 72.3 ± 15.5
Shah et al., 2016 [18]	n = 48 21 male	22 ± 2.5	175 ± 9.7 72.57 ± 1.29
Yvon et al., 2015 [29]	n = 50 13 male	NR	NR

cm = centimeter, kg = kilograms, n = sample size, NR = Not reported.

**Table 2:** Tasks and balance assessment protocol.

Author	Assessed Tasks	Feet Condition	Feet Position	Hands/Arms Position	Visual Input	Visual Reference
Alberts et al., 2015 [26]	Six conditions NeuroCom^®^ SOT	According to SOT	According to SOT	According to SOT	EO/EC	According to SOT
Alberts et al., 2015 [27]	Six conditions BESS	Wearing socks	According to BESS	Resting on the iliac crests	EC	NA
Kosse et al., 2015 [14]	Two conditions 1- Quiet standing 2- a Dual-task (letter fluency test)	NR	Parallel Semi-tandem	NR	EO/EC	NR
Hsieh et al., 2019 [30]	1- Quiet standing 2- a Dual-task (subtracting numbers)	Wearing socks	(NC ) Semi-tandem Tandem Single leg	dominate hand holding phone medially against the chest	EO/EC	NR
Ozinga et al., 2014 [15]	Six conditions adapted from BESS	Barefoot	According to BESS	Resting on the iliac crests	EO/EC	3m target
Patterson et al., 2014 [17]	Six conditions BESS (adapted) Five conditions *Sway Test*	Shoed	According to BESS	Holding Mobile at Sternum mid-point	EC	NA
Patterson et al., 2014 [28]	Single condition Athlete’s Single Leg Test	NR	Non-dominant foot stance	Holding Mobile at Sternum mid-point	EO	NR
Shah et al., 2016 [18]	Eight conditions	Barefoot	Apart Together Tandem	On the hips	EO/EC	4.37 m target
Yvon et al., 2015 [29]	Romberg and tandem Romberg tests in Sixteen conditions	NR	Apart Together Tandem	Side arms	EO/EC	NR

SOT = Sensory organization test, EO = Eyes open, EC = Closed eyes, BESS = Balance error scoring system, NA = Not applicable, NR = Not reported, NC = Not clearly stated.

**Table 3:** Balance protocol procedures, devices and technical specifications.

Author	Number of Trials	Total Time (Time Cropped) Seconds	Device I Device II (Sampling Rate)	Device Position	App Used for Acquisition	Synchronization
Alberts et al., 2015 [26]	3	20 s (NR)	iPad2 (100 Hz) NeuroCom^®^ (100 Hz)	Sacrum	Sensor Data by Wavefront Labs	LabVIEW data collection program.
Alberts et al., 2015 [27]	1	20 s (NR)	iPad (SNR) (100 Hz) Eagle 3D Motion analysis System (100 Hz)	Sacrum	Cleveland Clinic Concussion	Arduino Pro Mini 3.3 v and a LED light
Kosse et al., 2015 [14]	1	60 s (NR)	iPod Touch (88–92 Hz) Accelerometer DynaPort^®^ hybrid unit (100 Hz)	L3 vertebrae	iMoveDetection	Cross-correlation analysis
Hsieh et al., 2019 [30]	2	30 s (NR)	Samsung Galaxy S6 (200 Hz) Force plate (Bertec Inc, Columbus, OH) (1000 Hz)	Sternum	NR	NR
Ozinga et al., 2014 [15]	2	60 s (NR)	iPad 3 (100 Hz) Eagle 3D Motion analysis System (100 Hz)	Second sacral vertebrae	Cleveland Clinic Balance Assessment	Arduino Pro Mini 3.3 v and a LED light
Patterson et al., 2014 [17]	1	10 s STS 20 s BESS (NA)	iPod Touch (NR) NA	Sternum midpoint	SWAY Balance Mobile	NA
Patterson et al., 2014 [28]	1	10 s (NR)	iPod Touch (NR) Biodex^©^ Balance System (NR)	Sternum midpoint	SWAY Balance Mobile	NR
Shah et al., 2016 [18]	1	(NR)	LG Optimus One (14–15 Hz) NA	Malleols Patella Umbilics	myAnkle	NR
Yvon et al., 2015 [29]	1	30 s (NR)	iPhone (SNR) NA	Participant’s left upper arm	D + R Balance	NR

NR = Not reported, SNR = Specification of the device not reported, STS = Sway Test Software, NA= Not applicable, BESS = Balance error scoring system.

## Appendix A. Search Strategy (Databases)

MEDLINE accessed by Pubmed, Embase, Cochrane andSCOPUS up to February 2019.

The search strategy of the Pubmed, Embase, and SCOPUS databases was used the terms as follows:

#1
“Accelerometry”[Mesh]
“Accelerometer”
“Gyroscope”
“Bodywear sensors”
“Wearable sensors”
“Wear sensor”
“Inertial sensors”
“IMU”
“inertial measurement unit”
#2
“App Mobile”[Mesh]
“Apps, Mobile”
“Application, Mobile”
“Applications, Mobile”
“Mobile Application”
“Mobile Apps”
“Portable Electronic Apps”
“App, Portable Electronic”
“Apps, Portable Electronic”
“Electronic App, Portable”
“Electronic Apps, Portable”
“Portable Electronic App”
“Portable Electronic Applications”
“Application, Portable Electronic”
“Applications, Portable Electronic”
“Electronic Application, Portable”
“Electronic Applications, Portable”
“Portable Electronic Application”
“Portable Software Apps”
“App, Portable Software”
“Apps, Portable Software”
“Portable Software App”
“Software App, Portable”
“Software Apps, Portable”
“Portable Software Applications”
“Application, Portable Software”
“Applications, Portable Software”
“Portable Software Application”
“Software Application, Portable”
“Software Applications, Portable”
“mobile device”
“mobile smartphone”
“smartphone application”
“smartphone app”
#3
“Postural Balance”[MESH]
“Balance, Postural”
“Musculoskeletal Equilibrium”
“Equilibrium, Musculoskeletal”
“Postural Equilibrium”
“Equilibrium, Postural”
“Musculoskeletal Equilibrium”
“Equilibrium, Musculoskeletal”
“Postural Equilibrium”
“Equilibrium, Postural”
“Balance, Postural”
“sway”
“postural control”
“body sway”
#1 AND #2 AND #3

**Database:** Cochrane (up to February 2019).

“Accelerometer”OR“Mobile applications”AND“Postural Balance”.

## Appendix B. Quality Assessment Tool for Observational Cohort and Cross-Sectional Studies (NIH–NHLBI)

**Table A1:** Results of the quality assessment tool of the 9 studies (NIH-NHLBI).

Quality Assessment																
Study	1	2	3	4	5	6	7	8	9	10	11	12	13	14	Total	%
Alberts et al., 2015 [26]	2	2	2	2	0	0	0	2	2	2	2	-	-	0	16	67%
Alberts et al., 2015 [27]	2	2	2	2	2	0	0	2	0	0	2	-	-	2	16	67%
Kosse et al., 2015 [14]	2	2	2	2	2	0	0	2	0	2	2	-	-	0	16	67%
Hsieh et al., 2019 [30]	2	2	2	2	2	0	0	2	2	0	2	-	-	0	16	67%
Ozinga et al., 2014 [15]	2	2	2	2	2	0	0	2	2	0	2	-	-	2	18	75%
Patterson et al., 2014 [17]	2	2	2	2	2	0	0	2	0	0	1	-	-	0	13	50%
Patterson et al., 2014 [28]	2	2	2	2	2	0	0	0	0	0	2	-	-	0	12	50%
Shah et al., 2016 [18]	2	2	2	1	2	0	0	2	2	0	2	-	-	2	17	71%
Yvon et al., 2015 [29]	2	1	2	2	0	0	0	2	0	0	1	-	-	0	10	42%

The quality was expressed as a percentage of the total possible score, where each criterion could reach a maximum of two points (“Yes” = 2, Not Clear = “1”, “No” = 0). The studies were classified as: “high quality” (>75%), “moderate quality” (>50% to 75%), “low quality” (25% to 50%), and “very low quality” (<25%). Items 12 and 13 were considered not applicable due to the design characteristics of studies. Numbers 1 to 14 are related to each question of the quality assessment tool [25]

## Appendix C. 10-Point Checklist for Balance Assessment Protocols

**Table A2:** Results of the 10-Point Checklist for Balance Assessment tool of the 9 studies.

Author	Sample Information	Tasks Description	Feet Condition	Feet and Arms Position	Visual Reference Eyes	Visual Reference Target	Cropped Time	Sampling Rates	Data/Signal Processing Method	Synch Method	Total Core
Alberts et al., 2015 [26]	Y	Y	Y	Y	Y	Y	N	Y	Y	Y	9
Alberts et al., 2015 [27]	N	Y	Y	Y	Y	Y_(NA)	N	Y	Y	Y	8
Kosse et al., 2015 [14]	N	Y	N	N	Y	N	N	Y	Y	Y	5
Hsieh et al., 2019 [30]	N	Y	Y	N	Y	N	N	Y	Y	N	5
Ozinga et al., 2014 [15]	N	Y	Y	Y	Y	Y	N	Y	Y	Y	8
Patterson et al., 2014 [17]	Y	Y	Y	Y	Y	Y_(NA)	N	N	Y	Y_(NA)	8
Patterson et al., 2014 [28]	Y	Y	N	Y	Y	N	N	N	Y	N	5
Shah et al., 2016 [18]	Y	Y	Y	Y	Y	Y	N	Y	Y	N	8
Yvon et al., 2015 [29]	N	Y	N	Y	Y	N	N	N	N	Y_(NA)	4

Cropped time = Total time acquired *minus* Time window analyzed; *Synch* = Synchronization; Y = yes; N = no; Y_(NA) = for a “not applicable” item a “Y” was given.

## Appendix D. Overview (Description of Parameters and Measurements as Stated in the Study and General Comments)

**Table A3:** The primary signal processing parameters used in quantitative continuous data measurements as stated in the 9 studies.

Author	Overview	Parameters and Measurements	General Comments on the Strengths and Limitations
Alberts et al., 2015 [26]	Used the **Neurocom^®^ device** through force plate measurements with **sensory organization test** (SOT) to determine whether an iPad2 provides sufficient resolution of the center of gravity (COG) movements to quantify postural stability in healthy young people accurately.	Center of pressure (COP) of the anterior-posterior (AP) and medium-lateral (ML) sway. Three-dimensional (3D) device-rotation rates and linear acceleration. COG of the AP angle was used for all outcomes.	Only sample curves are shown for the CoG-AP sways for conditions 1, 4, 5 and 6. No numerical data are given for the actual physical measures from the Neurocom A-P sway-data as compared to the calculated A-P sway-data from iPad sensor. Nevertheless, the overall performance of the iPad for predicting the Equilibrium Score of the Neurocom appears excellent. The 100 Hz data sampling is more than sufficient to determine low-frequency body sways, probably using the smaller gadget/mobile, rather than the large iPad-2 may have resulted in even better results.
Alberts et al., 2015 [27]	Assessed the accuracy of the iPad by comparing the metrics of postural stability with a **3D motion capture system** and proposed a method of quantification of the Balance error scoring system (BESS) using the center of mass (COM) acceleration data.	3D Position, linear and angular accelerations of the COM at the AP and ML. 3D Linear acceleration and rotation-rate, (1) peak-to-peak, (2) normalized path length, (3) root mean square (RMS) of the displacements COM, (4) 95% ellipsoid volume of sway. A spectral analysis of ML, AP, and trunk (TR) acceleration.	No numerical data were compared between the motion capture results and the calculated values from the iPad sensors. Only correlations were calculated, and no raw data were presented, what leave readers not sure of the measurements’ consistency. This applies to Table 1, where no raw data for normalized path length (NPL), peak-to-peak (P2P), and RMS are presented, just comparisons with low to medium rho values. The small (Correlation coefficient) Rho values of only 0.55 and less (Table 2) for the iBESS Volume against the error score is even less convincing, but this can also be due to the low reliability of the subjective error scoring. The iPad sensors are probably much better able to detect balance deficits as compared to the more subjective BESS.
Kosse et al., 2015 [14]	Compared to the data from a stand-alone **accelerometer** unit to establish the validity and reliability of gait and posture assessment of an iPod.	AP and ML trunk acceleration and a resultant vector (1) RMS accelerations of body sway in AP and ML, (2) sway area, (3) total power median of the signal from frequency spectrum signals.	Good direct comparison study from an iPod with a "Gold Standard" DynaPort triaxial accelerometer. For comparing wave, similarity Cross-correlations were determined after time normalization (100 Hz). The values were around 0.9 for all experimental conditions in AP and ML directions suggesting a high-quality acceleration signal and software evaluation. Time-lag values were almost identical between the two transducers. Validity and test–retest reliability intraclass-correlations (ICC) values were also excellent for RMS signals in both the AP and ML direction. Only for the median power frequency (MPF) lower ICC´s were found for the test–retest reliability, (possibly caused by the different sampling frequencies, requiring time normalization procedures. Excellent and comprehensive analysis, including a measurement section for a pure comparison of transducer technology as well as application to groups of three age group participants.
Hsieh et al., 2019 [30]	Static balance tests were conducted while standing on a **force plate** and holding a smartphone. COP data from the force plate and acceleration data from the smartphone were compared. Validity between the measures was assessed and the Correlations coefficients were extracted to determine if a smartphone embedded accelerometer can measure static postural stability and distinguish older adults at high levels of fall risk.	The COP parameters included in the analysis were: (1) 95% confidence ellipse and (2) velocity in the anteroposterior (AP) direction and mediolateral (ML) direction. From the smartphone, (1) maximum acceleration in the ML, vertical, and AP directions and (4) root mean square (RMS) in the ML, vertical, and AP axis were exported and processed.	A promising approach was used to distinguish subjects with risks of falling associating acceleration data and COP parameters to the "physiological profile assessment” which is an evaluation of the risk of falling based on the assessment of multiple domains. Strong significant correlations between measures were found during challenging balance conditions (ρ = 0.42–0.81, *p* < 0.01–0.05). Correlations that, to some extent, were expected although it seems to be quite difficult to differentiate between vertical, AP, and ML components between the force plate and the accelerometer. Especially, during challenging balance tasks, there will be quite a bit of movement of the upper extremities against the body, creating all kinds of extra accelerations at the phone. A more trustful comparison of acceleration data from the phone to the force plate seems only possible if the phone would have been fastened at the CoG of subjects or close to the CoG.
Ozinga et al., 2014 [15]	Simultaneous kinematic measurements from a **3D motion analysis** system during balance conditions were used to compare the movements of COM to investigate if an iPad can provide sufficient accuracy and quality for the quantification of postural stability in older adults.	Angular velocities and linear accelerations were processed to allow direct comparison to Position of whole-body COM, (1) peak-to-peak displacement amplitude, (2) normalized path length, (3) RMS displacements of COM, (4) 95% ellipsoid volume of sway. Spectral analysis of the magnitude of the ML, AP, and trunk acceleration was used.	Fairly high correlations were present between the cinematographic, and the iPad derived data, suggesting that the iPad would be a good alternative to cinematographic posture analyses. Procedures and methods were well chosen. However, the number of subjects was fairly low.
Patterson et al., 2014 [17]	Compared the scores of a mobile technology application within an iPod through balance tasks with a commonly used subjective balance assessment, the **Balance error scoring system BESS**.	Balance scores by 3D Acceleration measurements.	An inverse relationship of r = -0.77 (*p* < 0.01) was found between the BEES score and the SWAY results. Thus, the iPod acceleration signals were proven to be a fairly good predictor of stability. An elevated BESS score reflected a high number of balance errors, whereas the SWAY Balance score assigns a higher value to more stable performance. The fact that subjects had to press the iPod with their hands against the sternum brings some weakness to the test procedures. It restricts freedom for balancing their body in the five exercises and introducing mechanical artifacts by having their hands at the sensors during balancing task. Because no direct comparisons between two sensors were made, only an indirect estimation of the quality of the iPod touch transducer can be made. Most likely, the major error of the limited r = -0.77 is not a function of the quality of the iPod sensor but rather a consequence of poor BESS rating quality. Mentioning yet, that BESS scoring test was done by only two people. However, since BESS is well accepted, this paper shows, that mobile phone integrated sensors are well suited for evaluating postural stability.
Patterson et al, 2014 [28]	A **Biodex© Balance System** which gives an AP Stability Index from a force platform was used to compare and evaluate the validity of a Balance Mobile Application which uses the 3D accelerometers from an iPod while subjects were performing a single trial of the Athlete Single Leg Test protocol.	Degree of tilt about each axis: (1) ML stability index, (2) AP stability index and the (3) overall stability index; The displacement in degrees from level was termed the “balance score” from the AP stability index (APSI).	AP stability index (APSI) score on the balance platform 1.41 was similar to the smartphone SWAY score 1.38 with no statistically significant difference. However, the correlation (ICC) between the scores was low - only r= 0.632 (p<0.01). As it was the case in the Patterson et al., 2014a, the same weakness was found, once the subjects had to hold the iPod touch with both hands at the sternum and only sway in AP direction was measured. Other than indicated the authors, an ICC of only r= 0.632 appears very low when considering that the same measure was taken by two systems at the same time for a single leg stance.
Shah et al, 2016 [18]	A mobile application was developed to provide a method of objectively measuring standing balance using the phone’s accelerometer. Eight independent therapists ranked a balance protocol based on their clinical experience to assess the degree of exercise difficulty. The concordance between the results was obtained to determine if the mobile can quantify standing balance and distinguish between exercises of varying difficulty.	3D accelerometer data were obtained from three mobile phones and mean acceleration was calculated; After a correction for static bias the corrected value was applied, the magnitude of the resultant vector (R) was calculated for each of measurement; The metric "mean R" was the average magnitude all resultant vectors and was then used as an index of balance.	Even though Shah et al., 2016 did not make a direct comparison between 2 sensor systems, accelerometer readings were calculated for each exercise at each ankle and knee and the torso. A high differentiation between the stability exercises shows lower values for ankle, knee, and torso, indicating that the acceleration results from the mobile phones have a strong relationship to the subjective rating of the 8 experienced clinicians. The results indicate that one sensor location appears sufficient since all sensors follow the same trend, it appears that knee, and torso locations could be used. From a practical point of view, easiest to mount and use would be the torso or hip location.
Yvon et al, 2015 [29]	An iPhone application was used to quantify sway while performing the Romberg and the Romberg tandem tests in a soundproof room and then in a normal room.	Output data (‘K’ value) was used to represent the area of an ellipse with two standard deviations in the anteriorposterior and lateral planes about a mean point.	The article explores a not usual protocol trying to evaluate the contributions of auditory sensory inputs on balance, through a combination of postures in different sound room condition. No raw data were presented or clearly specified; data processing procedures were not reported. Differences on postural sway measurements have been found among different room conditions with a dedicated application
